# Effect of Influenza Vaccination on Mortality and Risk of Hospitalization in Elderly Individuals with and without Disabilities: A Nationwide, Population-Based Cohort Study

**DOI:** 10.3390/vaccines8010112

**Published:** 2020-03-02

**Authors:** Yu-Chia Chang, Ho-Jui Tung, Yu-Tung Huang, Chin-Te Lu, Ernawaty Ernawaty, Szu-Yuan Wu

**Affiliations:** 1 Department of Healthcare Administration, College of Medical and Health Science, Asia University, Taichung 41354, Taiwan; ycchang@asia.edu.tw; 2 Department of Medical Research, China Medical University Hospital, China Medical University, Taichung 40402, Taiwan; 3 Department of Health Policy and Community Health, JPH College of Public Health, Georgia Southern University, Statesboro, GA 30458, USA; htung@georgiasouthern.edu; 4 Center for Big Data Analytics and Statistics, Chang Gung Memorial Hospital, Taoyuan 333, Taiwan; anton.huang@gmail.com; 5 Department of Infectious Diseases, Lo-Hsu Medical Foundation, Lotung Poh-Ai Hospital, Yilan 265, Taiwan; 964022@mail.pohai.org.tw; 6 Department of Health Policy and Administration, Faculty of Public Health, Universitas Airlangga, Surabaya 60115, Indonesia; ernawaty@fkm.unair.ac.id; 7 Division of Radiation Oncology, Lo-Hsu Medical Foundation, Lotung Poh-Ai Hospital, Yilan 265, Taiwan; 8 Big Data Center, Lo-Hsu Medical Foundation, Lotung Poh-Ai Hospital, Yilan 265, Taiwan; 9 Department of Food Nutrition and Health Biotechnology, College of Medical and Health Science, Asia University, Taichung 41354, Taiwan

**Keywords:** Influenza vaccination, elderly, disability, mortality, hospitalization

## Abstract

**Purpose**: The effects of influenza vaccines are unclear for elderly individuals with disabilities. We use a population-based cohort study to estimate the effects of influenza vaccines in elderly individuals with and without disabilities. **Methods**: Data were taken from the National Health Insurance Research Database and Disabled Population Profile of Taiwan. A total of 2,741,403 adults aged 65 or older were identified and 394,490 were people with a disability. These two groups were further divided into those who had or had not received an influenza vaccine. Generalized estimating equations (GEE) were used to compare the relative risks (RRs) of death and hospitalization across the four groups. **Results**: 30.78% elderly individuals without a disability and 34.59% elderly individuals with a disability had vaccinated for influenza. Compared to the unvaccinated elderly without a disability, the vaccinated elderly without a disability had significantly lower risks in all-cause mortality (RR = 0.64) and hospitalization for any of the influenza-related diseases (RR = 0.91). Both the unvaccinated and vaccinated elderly with a disability had significantly higher risks in all-cause mortality (RR = 1.81 and 1.18, respectively) and hospitalization for any of the influenza-related diseases (RR = 1.73 and 1.59, respectively). **Conclusions**: The elderly with a disability had higher risks in mortality and hospitalization than those without a disability; however, receiving influenza vaccinations could still generate more protection to the disabled elderly.

## 1. Introduction

Influenza remains a public health concern in Asia, and particularly in Taiwan [1]. The Taiwan Centers for Diseases Control (TCDC) and Infectious Disease Control Advisory Committee have multiple health strategies for seasonal influenza prevention in addition to the national influenza vaccination campaign for controlling the risk of influenza transmission among vulnerable groups and reducing influenza-associated mortality and hospitalization [1]. The influenza season in Taiwan usually begins in December and peaks in January or February of the following year [2]. Annual government-funded influenza vaccination campaigns aim to reduce the transmission of influenza and the associated mortality and morbidity [1,2,3]. Initially, Taiwan government-funded vaccines were only offered to adults ≥65 years [1,2,3,4,5]. Subsequently, people at high risk of developing serious complications and key spreaders, approximately 25% of the Taiwanese population, are vaccinated free of charge [1,2,4,5] However, individuals with a disability are not included in the current priority groups [6].

According to our previous studies using the National Health Insurance Research Database (NHIRD), influenza vaccines can reduce the risks of dementia, hemorrhage, and ischemic stroke in individuals with chronic kidney disease or atrial fibrillation [7,8,9]. However, few studies have demonstrated the observable benefits of influenza vaccine administration in elderly individuals with disabilities, especially in Asia. The current evidence is insufficient to establish recommendations for health policies. The question remains of whether influenza-related medical costs, hospitalizations, and mortality rates can be reduced in elderly individuals with disability through the administration of influenza vaccines. 

To explore the effects of influenza vaccines in elderly individuals with and without a disability, we used a retrospective cohort study built from NHIRD. The disability status of this population-based cohort was obtained through linking to the Disabled Population Profile of Taiwan, so that the effectiveness of the influenza vaccines can be compared between individuals with and without a disability. 

## 2. Methods

### 2.1. Data Source

In Taiwan, a universal insurance scheme called the National Health Insurance (NHI) was launched in 1995. The NHI is a national single-payer mandatory-enrollment health insurance program that covers more than 99.9% of the residents of Taiwan [8]. Data used in this study were retrieved from the NHIRD, provided by Taiwan’s National Health Insurance Administration and managed by the Health and Welfare Data Center, under the Ministry of Health and Welfare. This large computerized database contains all “insurance-covered” outpatient visits, hospitalizations, prescriptions, examinations, interventions, and related diagnoses. In this study, we used the NHIRD in combination with the Disabled Population Profile and the Cause of Death Registry. A detailed introduction to the NHIRD can be found in the literature [10].

In this population-based retrospective cohort study, data from 2014 on subjects aged 65 years or older were collected from the NHIRD. Disabilities were identified from the Disabled Population Profile, which contains data on all residents who claimed disability benefits. Disability certificates in Taiwan have been issued by accreditation of the national social welfare agency and professional physicians. Individuals with a disability certificate can enjoy many health value-added services and the benefits of social welfare units in Taiwan. Therefore, if an individual has a disability certificate, this is a financial burden of the Taiwan government. Thus, Taiwan government agencies do regular inspections. If a disability certificate is found to be false; its related social welfare institutions and the physicians will be guilty of criminal law. 

After excluding (1) those people who died before the end of 2014; (2) those people who received an influenza vaccine outside of the free influenza vaccination period (between 1 October and 31 December 2014); and (3) those who received more than one influenza vaccine during the study and follow-up period (between 1 January 2014, and 30 September 2015); a total of 394,490 individuals with a disability and 2,346,913 individuals without a disability who were aged 65 years or older in 2014, were included in the analysis. Data related to the identification of individuals were encrypted before being released to the researchers to protect personal privacy. The study was approved by the Chang Gung Medical Foundation Institutional Review Board (IRB No. 201900240B0).

### 2.2. Outcome Measures

Four outcome indicators, namely all-cause mortality, hospitalization, length of stay, and inpatient expenditure due to influenza-related complications during the influenza season were used to evaluate the effectiveness of the seasonal influenza vaccination. All-cause mortality was determined from the Cause of Death Registry. Hospitalizations, length of stay, and inpatient expenditures were determined from the NHIRD for hospital admissions due to influenza-related complications, including pneumonia (International Classification of Diseases, Ninth Revision, Clinical Modification [ICD-9-CM] codes 480–487), respiratory diseases (ICD-9-CM codes 460–466, 480–487, 490–496, and 500–518), chronic obstructive pulmonary disease (COPD; ICD9-CM codes 491, 492, and 496), respiratory failure (ICD-9-CM codes 518.81–518.84 and 799.1), heart diseases (ICD-9-CM codes 410–429), hemorrhagic stroke (ICD-9-CM 430, 431, and 432), and ischemic stroke (ICD-9-CM 433 and 434) [11,12].

The length of stay was measured as days hospitalized either in acute or chronic facilities. Inpatient expenditures were defined as the sum of inpatient medical expenses, including fees for physician and nurse care, surgeries or procedures, medications, exams and tests, and hospital stay as well as copayments and other miscellaneous fees [12].

The effectiveness of the influenza vaccination was evaluated using the outcome measures collected during peak influenza-like illness (ILI) activity [12,13], based on influenza-surveillance data from the TCDC. Compared with the previous five influenza seasons, the season of 2014–2015 was only moderately severe but lasted the longest. According to the surveillance data, ILI activity began to increase at the beginning of January 2015 and peaked in June, then plateaued at the end of September 2015 [14]. Therefore, the influenza season in this study was defined as the time period between January and September 2015. 

### 2.3. Measures of Predictors

The primary independent variable, whether the included individuals received the influenza vaccination, was determined by examining the NHIRD claims data (confirmed using drug codes) between 1 October 2014, and 31 December 2014, when the seasonal influenza vaccines were administered free of charge to adults aged 65 years or older in Taiwan. This variable is dichotomized and coded as 1 (received vaccination) and 0 (did not receive vaccination). 

Other baseline covariates were incorporated in this analysis, including sex, age, premium-based monthly salary, urbanization, Charlson comorbidity index (CCI) score, catastrophic illnesses, status as a long-term care facility resident, and healthcare utilization. The identified elderly were divided into four groups based on age: 65–69, 70–74, 75–79, and ≥80 years. Premium-based monthly salaries were divided into ≤NT$19,273, NT$19,274–NT$22,800; NT$22,801–NT$45,800; and ≥NT$45,801 (NT$ represents New Taiwan dollars). Urbanization of the area of residence was stratified into three levels: urban, suburban, and rural. The CCI scores were categorized as 0, 1–2, 3–4, and ≥5. Healthcare utilization included the number of outpatient visits, hospitalizations, and health examination over the course of 9 months prior to October 2014. The number of outpatient department (OPD) visits during the preceding 9 months was divided into two groups by the median visits of all elderly individuals aged 65 years and older in Taiwan (fewer than 18 visits versus more than 18 visits). 

### 2.4. Statistical Analysis

Bivariable comparisons of the covariates and influenza status were performed using chi-square tests. Four comparison groups were formed by cross-tabulating vaccination/no vaccination and disability/no disability. The generalized estimating equations (GEEs) method was used to compare the health outcomes for the four groups, measured between 1 January and 30 September 2015. The GEE logit estimated the effect of receiving influenza vaccination on the relative risks of death and hospitalization. The adjusted odds ratio was used as an estimate of relative risk (RR). Thus, the reduction in mortality and hospitalization was calculated as (1 − RR) × 100%. GEEs with a Poisson distribution were used to evaluate the length of stay. In addition, because the health care expenditure appeared to be right-skewed, the GEE model with a log-link and gamma distribution were incorporated. All analyses were performed using SAS version 9.4 (SAS Institute, Inc., Cary, North Carolina, USA). A two-tailed p-value of 0.05 was considered statistically significant.

### 2.5. Ethics Approval and Consent

Our protocols were reviewed and approved by the Chang Gung Medical Foundation Institutional Review Board (IRB No. 201900240B0). 

## 3. Results

The study cohort comprised elderly individuals without disability (*n* = 2,346,913), 722,317 (30.78%) of whom had received influenza vaccination and 1,624,596 (69.22%) of whom had not, and elderly individuals with disability (*n* = 394,490), 136,448 (34.59%) of whom had received influenza vaccination and the remaining 258,042 (65.41%) of whom had not (Table 1). Among the elderly individuals without disability, those who were male, older, having a lower premium salary, living in lower level of urbanization areas, having preexisting medical comorbidities, having a catastrophic illness, or residing in a long-term care facility had higher influenza vaccination rates. Additionally, people with higher outpatients visits, having been hospitalized, or having health examinations during the first 9 months of 2014 had higher influenza vaccination rates too. 

Among the elderly individuals with disability, those who were male, older, having a lower premium salary, having a catastrophic illness, residing in a long-term care facility, or having higher healthcare utilization had higher influenza vaccination rates. However, influenza vaccination rates were lower among those who living in more urbanized areas and having no comorbidities (see Table 1). In Table 2, when the individuals with disability were further divided into two groups, physical and mental disability, we found that the association patterns were the same as whole individuals with a disability presented in Table 1. Based on this, our subsequent analyses focus only on comparisons between the individuals with and without a disability.

Table 3 presents the GEE logit analysis results for the effect of receiving influenza vaccination on the RR of death and hospitalization. After adjustment for age, gender, premium salary, urbanization level, CCI score, catastrophic illness, status as a long-term care facility resident, and the number of outpatient visits, hospital admission, and utilization of health examination services, we analyzed the RR of death and hospitalization. Compared with the unvaccinated elderly without a disability, the RR of all-cause death was lower in vaccinated elderly individuals without a disability (RR = 0.64; 95% confidence interval (CI) = 0.63–0.66). Both unvaccinated and vaccinated elderly individuals with disability compared with unvaccinated elderly individuals without disability had significantly higher all-cause mortality rate (RR = 1.81; 95% CI = 1.78–1.84 and RR = 1.18; 95% CI = 1.16–1.21, respectively). 

However, vaccinated elderly individuals with a disability had a lower death risk than unvaccinated elderly individuals with a disability. The GEE analysis revealed that the RRs of hospitalization for influenza and pneumonia, respiratory diseases, respiratory failure, heart diseases, hemorrhagic stroke, and ischemic stroke, were significantly decreased in vaccinated elderly individuals without a disability when compared with the unvaccinated elderly without a disability. However, the RRs of hospitalization for influenza-related diseases were significantly higher in vaccinated (RR = 1.29–1.85) and unvaccinated (RR = 1.46–2.06) elderly individuals with a disability compared with unvaccinated elderly individuals without a disability. Similarly, vaccinated elderly individuals with a disability had a lower risk of hospitalization than unvaccinated elderly individuals with a disability (Table 3). 

The length of stay and medical expenditure, were compared between individuals with and without a disability, with and without IV, by using GEE, and the results are provided in Table 4. Among elderly individuals without a disability, the length of stay and medical expenditure during hospitalization were significantly lower in vaccinated individuals when compared with unvaccinated individuals. When compared with the unvaccinated elderly without a disability, the vaccinated and unvaccinated elderly with a disability were significantly associated with a higher length of stay (Exp (b) = 1.15 and 1.33, respectively) and medical expenditure (Exp (b) = 1.06 and 1.25, respectively) for any of the influenza-related disease hospitalizations (Table 4).

Overall, elderly individuals with a disability had an increased susceptibility to influenza-associated hospitalizations and death than elderly individuals without a disability, and those who were unvaccinated have a much greater risk than those who were vaccinated. However, vaccine administrations considerably reduced the rates of influenza-associated hospitalizations and deaths in elderly individuals with and without a disability. 

## 4. Discussion

The United States Advisory Committee on Immunization Practices advises that routine annual influenza vaccination is recommended for all persons aged ≥ 6 months without contraindications [15]. High-risk individuals, such as the elderly aged ≥65 years, patients with chronic diseases, children aged 6 through 59 months, those with pregnancy, etc.; their close contacts; and health care workers should remain high-priority populations in vaccination campaigns [15,16,17,18]. Prior to this study, little evidence suggested that influenza vaccines could reduce influenza-associated mortality and morbidity in elderly individuals with disabilities. 

The term “disability” in the Taiwan Disabled Population Profile refers to individuals with other chronic health conditions that interfere with or limit their physical, cognitive, or functional capacity [6]. It applies to people with neurological and neurodevelopmental conditions, such as disorders of the brain, spinal cord, peripheral nerves, and muscles; moderate to severe developmental delay; muscular dystrophy; and spinal cord injury [6]. For elderly individuals with a disability, their condition may affect their immune system, which in turn affects their capacity to fight off infections such as chronic and respiratory diseases, and thus puts them at increased risk of severe illness and need for hospitalization [6,19,20,21]. Additionally, they are at risk of influenza-associated mortality and morbidity due to limited mobility, they may have trouble understanding or practicing preventive measures, they may be unable to communicate symptoms of illness, and they may not be closely monitored for symptoms of illness [6,20,21]. Influenza vaccine administration is recommended for people at high risk of influenza-related complications; however, the effects of influenza vaccine administration in elderly individuals with disabilities have remained unknown. Therefore, in this study, we estimated and compared the effects of influenza vaccine administration in elderly individuals with and without disability.

Age [22], gender [22], salary [23], urbanization level [23], CCI score [24] preexisting major illness status [25], status as a long-term care facility resident [26], number of outpatient visits [26], hospital admission [27], and the utilization of health examination services [28] were associated with rates of influenza-related mortality and hospitalization. In this study, the vaccinated elderly individuals were more likely to be male, older, having a lower premium salary, having higher CCI scores, having a catastrophic illnesses, residing in a long-term care facility, having more outpatient visits, hospital admissions, and use of health examination services. Most of the covariates related to mortality and hospitalization due to influenza infection have been taken into account and the adjusted results are presented in Table 3 and Table 4. 

Our results showed that elderly individuals with disabilities have a higher rate of all-cause mortality, a longer duration of stay in hospitals, and a higher medical expenditure than elderly individuals without disabilities. Our results also showed that unvaccinated elderly individuals with a disability have higher rates of mortality and hospitalization than vaccinated elderly individuals with a disability (Table 3 and Table 4). Additionally, we further stratified the disability individuals into two groups, physical and mental disability, and repeated the analyses. The results (shown in Tables S1 and S2) indicate that the overall trends in individuals with a physical disability were similar to those with a mental disability. Thus, receiving influenza vaccines was associated with lower risks in all-cause mortality and influenza-related hospitalization, compared to those who did not vaccinate, regardless of their types of disability. It seems that receiving influenza vaccinations did generate more protection to all elderly individuals with disabilities.

The strength of our study was in having the largest cohort of elderly individuals with and without disability. To the best of our knowledge, no large cohort study has clearly demonstrated the effectiveness of influenza vaccines in elderly individuals with disabilities. This is the first population-based cohort study to demonstrate the decrease in all-cause mortality, influenza-related morbidity, the length of hospital stay, and medical expenditure in elderly individuals with disability as a result of influenza vaccination. This is also the leading study to demonstrate that elderly individuals with a disability were more susceptible to influenza-associated mortality and morbidity than elderly individuals without a disability, especially those who are not vaccinated have a much greater risk than those who are vaccinated. According to the results of our study, the government should closely monitor and encourage elderly individuals with disabilities to receive influenza vaccines regularly and the implementation strategies should be listed as national health policies. Furthermore, strengthened public health surveillance is needed. Special care for elderly individuals with disabilities can reduce health care costs and even the national financial burden in the future. 

This study has some limitations. First, as all individuals were of Asian ethnicity; therefore, our findings may not be suitable for extrapolation to non-Asian populations. However, influenza infection in Asians may be clinically similar to the disease in Caucasians [29]; the aim of future research is to compare the disease in the two populations. Second, the diagnoses of all comorbid conditions were based on ICD-9-CM codes. However, to verify the accuracy of the diagnoses, the Health and Welfare Data Center, under the Ministry of Health and Welfare Administration randomly reviews charts, interviews individuals, and ensures that hospitals with outlier chargers or practices are audited and subsequently heavily penalized if malpractice or discrepancies are identified. 

In addition, the quality and precision of ICD-9-CM codes in Taiwan have been verified and demonstrated by previous studies [30,31]. Therefore, we believe the conclusions of this study are reliable. Nevertheless, to obtain accurate information on population specificity and disease occurrence, a large-scale randomized trial comparing selected elderly individuals with disabilities who have or have not received an influenza vaccine is required. Finally, although comprehensive, the NHIRD lacks information, such as that concerning dietary habits or body mass index, which may be risk factors for influenza-related mortality.

## 5. Conclusions

Influenza vaccines reduce the rates of all-cause mortality and influenza-related hospitalization in elderly individuals with and without disabilities. Elderly individuals with a disability are more susceptible to mortality and hospitalization compared with unvaccinated elderly individuals without a disability. Health policies should encourage close monitoring of elderly individuals with a disability and the promotion of regular influenza vaccinations to reduce mortality and medical expenditure.

## Figures and Tables

**Table 1:** Baseline characteristics of all elderly individuals.

Variables	Total (N = 2,741,403)		Elderly Individuals without Disability (N = 2,346,913)			Elderly Individuals with Disability (N = 394,490)		
	*n*	%	Without IV	With IV	*p*-Value ^1^	Without IV	With IV	*p*-Value ^1^
			%	%		%	%	
Total	2,741,403	100.00	1,624,596 (69.22%)	722,317 (30.78%)		258,042 (65.41%)	136,448 (34.59%)	
Gender					<0.001			<0.001
Female	1,469,819	53.62	69.75	30.25		66.18	33.82	
Male	1,271,584	46.38	68.60	31.40		64.64	35.36	
Age (year) (mean ± SD)	74.53 ± 7.33		73.51 ± 7.16	75.39 ± 6.95	<0.001	76.76 ± 8.01	77.90 ± 7.56	<0.001
65–69	840,533	30.66	77.77	22.23		72.91	27.09	
70–74	683,448	24.93	69.33	30.67		66.99	33.01	
75–79	529,462	19.31	62.59	37.41		62.55	37.45	
≧80	687,960	25.10	62.54	37.46		62.12	37.88	
Premium salary (NTD)					<0.001			<0.001
19,273	884,340	32.26	71.19	28.81		65.70	34.30	
19,274-22,800	1,098,738	40.08	65.17	34.83		63.29	36.71	
22,801-45,800	461,607	16.84	73.31	26.69		68.89	31.11	
≧45,801	296,718	10.82	71.87	28.13		67.76	32.24	
Urbanization					<0.001			<0.001
Urban	1,453,747	53.03	73.12	26.88		68.46	31.54	
Suburban	893,278	32.58	65.94	34.06		63.43	36.57	
Rural	394,378	14.39	61.80	38.20		60.75	39.25	
CCI score					<0.001			<0.001
0	2,170,934	79.19	70.67	29.33		67.43	32.57	
1 or 2	368,417	13.44	62.69	37.31		61.86	38.14	
3 or 4	147,741	5.39	62.33	37.67		61.41	38.59	
≧5	54,311	1.98	64.54	35.46		62.64	37.36	
Catastrophic illnesses					<0.001			<0.001
No	2,480,370	90.48	69.37	30.63		65.70	34.30	
Yes	261,033	9.52	67.45	32.55		64.35	35.65	
Long-term care facility resident					<0.001			<0.001
No	2,713,653	98.99	69.32	30.68		66.73	33.27	
Yes	27,750	1.01	43.10	56.90		39.73	60.27	
Utilization of outpatient care ^2^					<0.001			<0.001
<18	1,370,072	49.98	76.41	23.59		75.28	24.72	
≧18	1,371,331	50.02	61.39	38.61		59.62	40.38	
Hospital admission ^2^					<0.001			<0.001
No	2,357,683	86.00	69.51	30.49		65.83	34.17	
Yes	383,720	14.00	67.12	32.88		64.16	35.84	
Utilization of health examination services ^2^					<0.001			<0.001
No	1,969,323	71.84	73.88	26.12		69.85	30.15	
Yes	772,080	28.16	57.85	42.15		50.45	49.55	

^1^ Chi-square test; ^2^ Utilization status during the preceding 9 months; Abbreviations: IV, influenza vaccine; CCI, Charlson Comorbidity Index; NTD, New Taiwan dollar.

**Table 2:** Baseline characteristics of all disability elderly individuals.

Variables	Elderly Individuals with All Disability (N = 394,490)		Elderly Individuals with Physical Disability ^1^ (N = 346,176)			Elderly Individuals with Mental Disability ^2^ (N = 48,314)		
	*n*	%	Without IV	With IV	*p*-Value ^3^	Without IV	With IV	*p*-Value ^3^
			%	%		%	%	
Total	394,490	100.00	227,157 (65.62%)	119,019 (34.38%)		30,885 (63.93%)	17,429 (36.07%)	
Gender					<0.001			<0.001
Female	198,020	50.20	66.46	33.54		64.62	35.38	
Male	196,470	49.80	64.83	35.17		62.72	37.28	
Age (year) (mean ± SD)	77.15 ± 7.88		76.60 ± 7.92	77.73 ± 7.46	<0.001	77.92 ± 8.60	79.00 ± 8.16	<0.001
65–69	80,224	20.34	73.15	26.85		71.17	28.83	
70–74	81,779	20.73	67.24	32.76		64.69	35.31	
75–79	80,924	20.51	62.61	37.39		61.99	38.01	
≧80	151,563	38.42	62.27	37.73		61.27	38.73	
Premium salary (NTD)					<0.001			<0.001
19,273	133,071	33.73	66.12	33.88		63.52	36.48	
19,274-22,800	161,451	40.93	63.31	36.69		63.07	36.93	
22,801-45,800	61,481	15.58	69.22	30.78		66.21	33.79	
≧45,801	38,487	9.76	68.12	31.88		65.19	34.81	
Urbanization					<0.001			<0.001
Urban	191,671	48.59	68.77	31.23		66.49	33.51	
Suburban	134,518	34.10	63.69	36.31		61.40	38.60	
Rural	68,301	17.31	60.85	39.15		59.86	40.14	
CCI score					<0.001			<0.001
0	252,010	63.88	67.65	32.35		66.09	33.91	
1 or 2	81,150	20.57	62.36	37.64		57.66	42.34	
3 or 4	41,112	10.42	61.75	38.25		57.37	42.63	
≧5	20,218	5.13	62.66	37.34		62.14	37.86	
Catastrophic illnesses					<0.001			<0.001
No	310,708	78.76	65.85	34.15		64.44	35.56	
Yes	83,782	21.24	64.69	35.31		62.88	37.12	
Long-term care facility resident					<0.001			<0.001
No	375,171	95.10	66.80	33.20		66.25	33.75	
Yes	19,319	4.90	40.09	59.91		38.39	61.61	
Utilization of outpatient care ^4^					<0.001			<0.001
<18	145,799	36.96	75.27	24.73		75.34	24.66	
≧18	248,691	63.04	60.16	39.84		55.30	44.70	
Hospital admission ^4^					<0.001			<0.001
No	295,965	75.02	65.92	34.08		65.17	34.83	
Yes	98,525	24.98	64.71	35.29		60.38	39.62	
Utilization of health examination services ^4^					<0.001			<0.001
No	304,258	77.13	69.89	30.11		69.55	30.45	
Yes	90,232	22.87	51.38	48.62		43.34	56.66	

^1^ Physical disabilities included visual impairment, hearing impairment, sound and speech impairment, physical disability, multiple disabilities, major organ malfunction, facial injury, balance impairment, refractory epilepsy, persistent vegetative state, and rare diseases. ^2^ Mental disabilities included intellectual impairment, dementia, autism, chromosomal abnormalities, metabolic abnormalities, congenital defects, and psychiatric disorders; ^3^ Chi-square test; ^4^ Utilization status during the preceding 9 months; Abbreviations: IV, influenza vaccine; CCI, Charlson Comorbidity Index; NTD, New Taiwan dollar.

**Table 3:** Comparison of the risk of outcomes between elderly individuals according to the presence of a disability and according to whether a vaccine was received.

Outcomes	Total (N = 2,741,403)	Without Disability/ without IV (ref.) (N = 1,624,596)	Without Disability/with IV(N = 722,317)					With Disability/without IV(N = 258,042)					With Disability/with IV(N = 136,448)				
	Incident (‰)	Incident (‰)	Incident (‰)	RR ^1^	95% CI			Incident (‰)	RR ^1^	95% CI			Incident (‰)	RR ^1^	95% CI		
**All-cause death**	34.06	27.10	22.05	0.64***	0.63	-	0.66	88.96	1.81***	1.78	-	1.84	76.59	1.18***	1.16	-	1.21
**Hospitalization**																	
Influenza or pneumonia	38.00	26.59	30.45	0.88***	0.86	-	0.89	91.99	2.01***	1.97	-	2.04	111.84	1.85***	1.81	-	1.90
Respiratory diseases	58.06	42.55	48.88	0.89***	0.87	-	0.90	131.09	1.89***	1.86	-	1.92	153.19	1.72***	1.69	-	1.75
COPD	21.35	15.15	21.29	1.02	1.00	-	1.04	41.02	1.46***	1.42	-	1.49	58.33	1.56***	1.52	-	1.61
Respiratory failure	15.76	11.04	11.42	0.81***	0.79	-	0.83	42.51	2.06***	2.01	-	2.12	44.32	1.63***	1.58	-	1.69
Heart disease	44.33	35.82	41.79	0.95***	0.94	-	0.97	81.26	1.47***	1.45	-	1.50	89.17	1.39***	1.36	-	1.42
Hemorrhagic stroke	2.23	1.99	1.81	0.82***	0.77	-	0.87	4.11	1.54***	1.44	-	1.66	3.65	1.30***	1.17	-	1.43
Ischemic stroke	9.01	8.01	7.88	0.85***	0.83	-	0.88	15.61	1.50***	1.44	-	1.55	14.35	1.29***	1.23	-	1.36
Any of the above disease	87.16	68.16	77.69	0.91***	0.90	-	0.92	174.77	1.73***	1.70	-	1.75	197.81	1.59***	1.56	-	1.62

^1^ Abbreviations: ref., reference group; IV, influenza vaccine; RR, relative risk; COPD, Chronic obstructive pulmonary disease; CI, confidence interval; ^2^ All models were analyzed using the generalized estimating equation. Extraneous factors adjusted in the model were age, gender, premium salary, urbanization level, CCI score, catastrophic illnesses, status as a long-term care facility resident, outpatient utilization, hospital admission, and utilization of health examination services. * *p* < 0.05; ** *p* < 0.01; *** *p* < 0.001.

**Table 4:** Comparing the length of stay and medical expenditure between elderly individuals according to the presence of a disability and according to whether the vaccine was received.

Outcomes	Without Disability/without IV (ref.)	Without Disability/with IV					With Disability/without IV					With Disability/with IV				
	Mean	Mean	Exp (b)	95% CI			Mean	Exp (b)	95% CI			Mean	Exp (b)	95% CI		
**Length of stay**																
Influenza or pneumonia	19.36	19.01	0.95***	0.95	-	0.96	24.18	1.17***	1.17	-	1.18	22.61	1.05***	1.05	-	1.06
Respiratory diseases	20.82	19.71	0.93***	0.93	-	0.94	27.40	1.23***	1.23	-	1.24	24.62	1.07***	1.07	-	1.08
COPD	16.03	15.66	0.95***	0.95	-	0.96	23.25	1.33***	1.32	-	1.33	20.85	1.14***	1.13	-	1.15
Respiratory failure	31.94	30.92	0.98***	0.97	-	0.98	38.65	1.20***	1.20	-	1.21	33.04	1.04***	1.03	-	1.04
Heart disease	13.12	11.95	0.91***	0.91	-	0.91	17.09	1.22***	1.21	-	1.22	15.39	1.08***	1.07	-	1.09
Hemorrhagic stroke	25.32	25.09	0.93***	0.92	-	0.94	25.65	1.04***	1.02	-	1.05	21.19	0.89***	0.87	-	0.91
Ischemic stroke	17.97	16.02	0.91***	0.90	-	0.92	18.98	1.06***	1.05	-	1.07	16.55	0.94***	0.93	-	0.96
Any of the above disease	39.04	37.32	0.92***	0.92	-	0.92	58.38	1.33***	1.33	-	1.34	53.93	1.15***	1.15	-	1.15
**Medical expenditure**																
Influenza or pneumonia	127,283	118,958	0.93***	0.91	-	0.95	152,579	1.13***	1.11	-	1.15	133,165	0.98*	0.96	-	1.00
Respiratory diseases	144,588	131,018	0.92***	0.91	-	0.93	173,649	1.14***	1.13	-	1.16	148,062	0.98 *	0.96	-	1.00
COPD	90,824	86,951	0.95***	0.93	-	0.97	119,238	1.21***	1.18	-	1.24	105,366	1.06 ***	1.03	-	1.09
Respiratory failure	273,615	259,611	0.98	0.95	-	1.01	285,946	1.06***	1.03	-	1.08	243,127	0.94***	0.91	-	0.97
Heart disease	116,230	106,596	0.94***	0.93	-	0.96	131,848	1.10***	1.08	-	1.12	116,959	1.00	0.98	-	1.03
Hemorrhagic stroke	207,191	208,435	0.93***	0.92	-	0.94	201,859	0.99	0.92	-	1.07	156,093	0.82***	0.74	-	0.92
Ischemic stroke	104,378	98,026	0.95**	0.92	-	0.98	115,741	1.09***	1.05	-	1.13	101,478	0.97	0.92	-	1.02
Any of the above disease	283,865	263,189	0.91***	0.90	-	0.93	384,479	1.25***	1.23	-	1.26	338,457	1.06***	1.04	-	1.08

Abbreviations: ref., reference group; IV, influenza vaccine; COPD, chronic obstructive pulmonary disease; ^1^ All models were analyzed using the generalized estimating equation. Extraneous factors adjusted in the model were age, gender, premium salary, urbanization level, CCI score, catastrophic illnesses, status as long-term care facility resident, outpatient utilization, hospital admission, and utilization of health examination services. * *p* < 0.05; ** *p* < 0.01; *** *p* < 0.001.

## Supplementary Materials

The following are available online at https://www.mdpi.com/2076-393X/8/1/112/s1, Table S1: Comparison of the risk of outcomes according to the presence of a physical disability and according to whether or not the vaccine was received. Table S2: Comparison of the risk of outcomes according to the presence of a mental disability and according to whether or not the vaccine was received.

## Abbreviations

NHIRD: National Health Insurance Research Database; GEE, Generalized estimating equations; RR, relative risk; TCDC, Taiwan Centers for Diseases Control; IDCAC, Infectious Disease Control Advisory Committee; NHI, National Health Insurance; ICD-9-CM, International Classification of Diseases, Ninth Revision, Clinical Modification; ILI, influenza-like illness; CCI, Charlson Comorbidity Index; NTD, New Taiwan dollars; OPD, outpatient department; ACIP, Advisory Committee on Immunization Practices.

## References

[1] Su C.P., Tsou T.P., Chen C.H., Lin T.Y., Chang S.C. (2019). Seasonal influenza prevention and control in Taiwan-Strategies revisited. J. Formos. Med. Assoc..

[2] Chuang J.H., Huang A.S., Huang W.T., Liu M.T., Chou J.H., Chang F.Y., Chiu W.T. (2012). Nationwide surveillance of influenza during the pandemic (2009–2010) and post-pandemic (2010–2011) periods in Taiwan. PLoS ONE.

[3] National Vaccine Advisory Committee (2013). Strategies to achieve the healthy people 2020 annual influenza vaccine coverage goal for health-care personnel: Recommendations from the national vaccine advisory committee. Public Health Rep..

[4] Wang S.T., Lee L.T., Chen L.S., Chen T.H. (2005). Economic evaluation of vaccination against influenza in the elderly: An experience from a population-based influenza vaccination program in Taiwan. Vaccine.

[5] Lo Y.C., Chuang J.H., Kuo H.W., Huang W.T., Hsu Y.F., Liu M.T., Chen C.H., Huang H.H., Chang C.H., Chou J.H. (2013). Surveillance and vaccine effectiveness of an influenza epidemic predominated by vaccine-mismatched influenza B/Yamagata-lineage viruses in Taiwan, 2011–2012 season. PLoS ONE.

[6] Chang Y.C., Tung H.J., Hsu S.W., Chen L.S., Kung P.T., Huang K.H., Chiou S.J., Tsai W.C. (2016). Use of Seasonal Influenza Vaccination and Its Associated Factors among Elderly People with Disabilities in Taiwan: A Population-Based Study. PLoS ONE.

[7] Liu J.C., Hsu Y.P., Kao P.F., Hao W.R., Liu S.H., Lin C.F., Sung L.C., Wu S.Y. (2016). Influenza Vaccination Reduces Dementia Risk in Chronic Kidney Disease Patients: A Population-Based Cohort Study. Medicine.

[8] Liu J.C., Wang T.J., Sung L.C., Kao P.F., Yang T.Y., Hao W.R., Chen C.C., Hsu Y.P., Wu S.Y. (2017). Influenza vaccination reduces hemorrhagic stroke risk in patients with atrial fibrillation: A population-based cohort study. Int J. Cardiol..

[9] Kao P.F., Liu J.C., Hsu Y.P., Sung L.C., Yang T.Y., Hao W.R., Lin Y.C., Wu S.Y. (2017). Influenza vaccination might reduce the risk of ischemic stroke in patients with atrial fibrillation: A population-based cohort study. Oncotarget.

[10] Hsieh C.Y., Su C.C., Shao S.C., Sung S.F., Lin S.J., Kao Yang Y.H., Lai E.C. (2019). Taiwan’s National Health Insurance Research Database: Past and future. Clin. Epidemiol..

[11] Nichol K.L., Nordin J., Mullooly J., Lask R., Fillbrandt K., Iwane M. (2003). Influenza vaccination and reduction in hospitalizations for cardiac disease and stroke among the elderly. N. Engl. J. Med..

[12] Chang Y.C., Chou Y.J., Liu J.Y., Yeh T.F., Huang N. (2012). Additive benefits of pneumococcal and influenza vaccines among elderly persons aged 75 years or older in Taiwan--a representative population-based comparative study. J. Infect..

[13] Tsai Y.W., Huang W.F., Wen Y.W., Chen P.F. (2007). The relationship between influenza vaccination and outpatient visits for upper respiratory infection by the elderly in Taiwan. Value Health.

[14] Taiwan Centers for Disease Control (2016). Statistics of Communicable Diseases and Surveillance Report 2015.

[15] Grohskopf L.A., Sokolow L.Z., Broder K.R., Walter E.B., Fry A.M., Jernigan D.B. (2018). Prevention and Control of Seasonal Influenza with Vaccines: Recommendations of the Advisory Committee on Immunization Practices—United States, 2018–2019 Influenza Season. MMWR Recomm. Rep..

[16] Monto A.S. (2010). Seasonal influenza and vaccination coverage. Vaccine.

[17] Sullivan S.J., Jacobson R., Poland G.A. (2010). Advances in the vaccination of the elderly against influenza: Role of a high-dose vaccine. Exp. Rev. Vaccines.

[18] Grohskopf L.A., Alyanak E., Broder K.R., Walter E.B., Fry A.M., Jernigan D.B. (2019). Prevention and Control of Seasonal Influenza with Vaccines: Recommendations of the Advisory Committee on Immunization PracticesUnited States, 2019–2020 Influenza Season. MMWR Recomm. Rep..

[19] Nichol K.L. (2009). Challenges in evaluating influenza vaccine effectiveness and the mortality benefits controversy. Vaccine.

[20] Bocquier A., Fressard L., Paraponaris A., Davin B., Verger P. (2017). Seasonal influenza vaccine uptake among people with disabilities: A nationwide population study of disparities by type of disability and socioeconomic status in France. Prev. Med..

[21] Garibaldi R.A., Nurse B.A. (1986). Infections in the elderly. Am. J. Med..

[22] Quandelacy T.M., Viboud C., Charu V., Lipsitch M., Goldstein E. (2014). Age- and sex-related risk factors for influenza-associated mortality in the United States between 1997–2007. Am. J. Epidemiol..

[23] Armstrong K., Berlin M., Schwartz J.S., Propert K., Ubel P.A. (2001). Barriers to influenza immunization in a low-income urban population. Am. J. Prev. Med..

[24] Shih C.H., Lee Y.J., Chao P.W., Kuo S.C., Ou S.M., Huang H.M., Chen Y.T. (2018). Association between influenza vaccination and the reduced risk of acute kidney injury among older people: A nested case-control study. Eur. J. Int. Med..

[25] Voordouw B.C., van der Linden P.D., Simonian S., van der Lei J., Sturkenboom M.C., Stricker B.H. (2003). Influenza vaccination in community-dwelling elderly: Impact on mortality and influenza-associated morbidity. Arch. Int. Med..

[26] Gaillat J., Chidiac C., Fagnani F., Pecking M., Salom M., Veyssier P., Carrat F. (2009). Morbidity and mortality associated with influenza exposure in long-term care facilities for dependent elderly people. Eur. J. Clin. Microbiol. Infect. Dis..

[27] Simonsen L., Fukuda K., Schonberger L.B., Cox N.J. (2000). The impact of influenza epidemics on hospitalizations. J. Infect. Dis..

[28] Pham H.H., Schrag D., Hargraves J.L., Bach P.B. (2005). Delivery of preventive services to older adults by primary care physicians. JAMA.

[29] Srivastav A., O’Halloran A., Lu P.J., Williams W.W. (2018). Influenza Vaccination Coverage Among English-Speaking Asian Americans. Am. J. Prev. Med..

[30] Cheng C.L., Lee C.H., Chen P.S., Li Y.H., Lin S.J., Yang Y.H. (2014). Validation of acute myocardial infarction cases in the national health insurance research database in taiwan. J. Epidemiol..

[31] Hsing A.W., Ioannidis J.P. (2015). Nationwide Population Science: Lessons From the Taiwan National Health Insurance Research Database. JAMA Int. Med..

