# Supplementary material for: Effect of Influenza Vaccination on Mortality and Risk of Hospitalization in Elderly Individuals with and without Disabilities: A Nationwide, Population-Based Cohort Study
Source: Vaccines (Basel). 2020 Mar 2;8(1):112. doi: 10.3390/vaccines8010112 (PMC7157235; doi:10.3390/vaccines8010112)
Supplement: Supplementary file 1 [file vaccines-08-00112-s001.pdf]

Supplemental Table S1. Comparison of the risk of outcomes according to the presence of physical disability and according to whether or not the vaccine was received

	Total (N=2,741,403)	Without disability/ without IV (ref.) (N=1,624,596)	Without disability/with IV (N=722,317)			Physical disability/without IV (N=227,157)			Physical disability/with IV (N=119,019)		
**Outcomes**	**Incident (‰)**	**Incident (‰)**	**Incident (‰)**	**RR[1]**	**95% CI**	**Incident (‰)**	**RR[1]**	**95% CI**	**Incident (‰)**	**RR[1]**	**95% CI**
**All-cause death**	34.06	27.10	22.05	0.64***	0.63 - 0.66	87.68	1.78***	1.75 - 1.81	74.20	1.15***	1.12 - 1.18
**Hospitalization**											
Influenza or pneumonia	38.00	26.59	30.45	0.88***	0.86 - 0.89	89.54	1.93***	1.89 - 1.97	105.82	1.73***	1.69 - 1.77
Respiratory diseases	58.06	42.55	48.88	0.89***	0.87 - 0.90	128.73	1.83***	1.80 - 1.86	146.83	1.62***	1.59 - 1.66
COPD	21.35	15.15	21.29	1.02	1.00 - 1.04	40.71	1.42***	1.39 - 1.46	56.25	1.50***	1.45 - 1.54
Respiratory failure	15.76	11.04	11.42	0.81***	0.79 - 0.83	41.99	2.03***	1.97 - 2.08	42.76	1.58***	1.52 - 1.63
Heart disease	44.33	35.82	41.79	0.95***	0.94 - 0.97	83.45	1.50***	1.47 - 1.53	91.17	1.42***	1.39 - 1.45
Hemorrhagic stroke	2.23	1.99	1.81	0.82***	0.77 - 0.87	4.05	1.50***	1.39 - 1.62	3.65	1.27***	1.14 - 1.41
Ischemic stroke	9.01	8.01	7.88	0.85***	0.83 - 0.88	15.78	1.49***	1.44 - 1.55	14.48	1.28***	1.22 - 1.35
Any of the above disease	87.16	68.16	77.69	0.91***	0.90 - 0.92	174.03	1.70***	1.68 - 1.72	193.64	1.54***	1.52 - 1.57

[1] Abbreviations: ref., reference group; IV, influenza vaccine; RR, relative risk; COPD, Chronic obstructive pulmonary disease; CI, confidence interval

[2] All models were analyzed using the generalized estimating equation. Extraneous factors adjusted in the model were age, gender, premium salary, urbanization level, CCI score, catastrophic illnesses, status as a long-term care facility resident, outpatient utilization, hospital admission, and utilization of health examination services.

*p<0.05; **p<0.01; ***p<0.001

Supplemental Table S2. Comparison of the risk of outcomes according to the presence of mental disability and according to whether or not the vaccine was received

	**Total (N=2,741,403)**	**Without disability/ without IV (ref.) (N=1,624,596)**	**Without disability/with IV (N=722,317)**			**Mental disability/without IV (N=30,885)**			**Mental disability/with IV (N=17,429)**		
**Outcomes**	**Incident (‰)**	**Incident (‰)**	**Incident (‰)**	**RR[1]**	**95% CI**	**Incident (‰)**	**RR[1]**	**95% CI**	**Incident (‰)**	**RR[1]**	**95% CI**
**All-cause death**	34.06	27.10	22.05	0.64***	0.63 - 0.66	98.40	1.77***	1.70 - 1.85	92.89	1.17***	1.10 - 1.24
**Hospitalization**											
Influenza or pneumonia	38.00	26.59	30.45	0.88***	0.86 - 0.89	110.05	2.38***	2.29 - 2.48	152.96	2.39***	2.28 - 2.51
Respiratory diseases	58.06	42.55	48.88	0.89***	0.87 - 0.90	148.45	2.21***	2.13 - 2.29	196.63	2.18***	2.09 - 2.28
COPD	21.35	15.15	21.29	1.02	1.00 - 1.04	43.32	1.64***	1.55 - 1.74	72.52	1.93***	1.80 - 2.06
Respiratory failure	15.76	11.04	11.42	0.81***	0.79 - 0.83	46.33	2.05***	1.93 - 2.17	54.97	1.64***	1.53 - 1.77
Heart disease	44.33	35.82	41.79	0.95***	0.94 - 0.97	65.11	1.20***	1.14 - 1.26	75.51	1.12***	1.05 - 1.19
Hemorrhagic stroke	2.23	1.99	1.81	0.82***	0.77 - 0.87	4.57	1.78***	1.50 - 2.11	3.67	1.36*	1.05 - 1.75
Ischemic stroke	9.01	8.01	7.88	0.85***	0.83 - 0.88	14.34	1.43***	1.29 - 1.57	13.48	1.24**	1.09 - 1.42
Any of the above disease	87.16	68.16	77.69	0.91***	0.90 - 0.92	180.25	1.84***	1.78 - 1.90	226.29	1.79***	1.72 - 1.86

[1] Abbreviations: ref., reference group; IV, influenza vaccine; RR, relative risk; COPD, Chronic obstructive pulmonary disease; CI, confidence interval

[2] All models were analyzed using the generalized estimating equation. Extraneous factors adjusted in the model were age, gender, premium salary, urbanization level, CCI score, catastrophic illnesses, status as a long-term care facility resident, outpatient utilization, hospital admission, and utilization of health examination services.

*p<0.05; **p<0.01; ***p<0.001
